# Annuity payments can increase patient access to innovative cell and gene therapies under England’s net budget impact test

**DOI:** 10.1080/20016689.2017.1355203

**Published:** 2017-07-31

**Authors:** Jesper Jørgensen, Panos Kefalas

**Affiliations:** ^a^ Cell Therapy Catapult Limited, Guy’s Hospital, London, UK

**Keywords:** Budget impact, pricing and reimbursement, patient access, managed entry agreement, risk-sharing, high-cost, advanced therapy medicinal product (ATMP), cell therapy, gene therapy

## Abstract

**Background:** Cell and gene therapies have the potential to provide therapeutic breakthroughs, but the high costs of researching, developing, manufacturing and delivering them translate into prices that may challenge healthcare budgets. Various measures exist that aim to address the affordability challenge, including reducing price, limiting patient numbers and/or linking remuneration to product performance.

**Objective:** To explore how the net budget impact test recently introduced in England can affect patient access to high-value, one-off cell and gene therapies, and how managed entry agreements can improve access.

**Methods:** We use a hypothetical example where a new high-value, one-off therapy launches in an indication where it displaces a relatively low cost chronic treatment. We calculate the number of patients that can be treated without exceeding the £20 million net budget impact threshold, and compare results for scenarios where a full upfront payment is used, and where annuity-based payments are used.

**Results**: Charging a full upfront payment at the time of treatment can lead to suboptimal patient access.

**Conclusion:** Annuity-based payments in combination with an outcomes-based remuneration scheme reduce consequences of decision uncertainty and can increase patient access, without exceeding the net budget impact test.

## Introduction and objectives

Cell and gene therapies belong to a category of treatments called advanced therapy medicinal products (ATMPs), which under European regulation encompasses gene therapies, somatic cell therapies and tissue-engineered products [1]. ATMPs are innovative therapies that combine aspects of medicine, cell biology, science and engineering for the purpose of regenerating, repairing or replacing damaged tissues or cells [2], and have the potential to offer substantial improvements in therapeutic benefit [3]. A key distinguishing feature of ATMPs, as compared to conventional pharmaceuticals (e.g., orals), is that ATMPs are more complex to research, develop, manufacture and deliver. These complexities increase costs, which has a knock-on effect on the price that manufacturers need to charge in order to ensure commercial viability and continued research and development (R&D).

When managing constrained healthcare budgets, policymakers face the challenge of striking a balance between maximising patient benefit while incentivising the industry to research and develop innovative therapies in areas of unmet need, and ensuring affordability and sustainability of healthcare funding. Several measures exist that aim to address the affordability challenge healthcare systems face, including cost-based measures, volume-based measures, and outcomes-based measures. In March 2017, a budget impact test was introduced in England, which assesses whether a new therapy’s aggregate additional cost to the healthcare budget exceeds the threshold value of £20 million per year. If the additional cost associated with the new therapy is expected to exceed this threshold in any of the first three years after launch, then additional commercial negotiations and potential restrictions apply [4].

This budget impact test means that the higher the net budget impact per patient is for a new therapy, the fewer patients can be treated with it in order not to exceed the £20 million threshold. This has the potential to lead to a situation where patient access to high-value treatments is compromised. The objective of this article is to explore how the net budget impact test can affect patient access to new, high-value, one-off ATMPs, and how access can be improved by using outcomes-based annuity payments.

## Background

### Balancing value for money and affordability for healthcare systems

The launch of several high-cost medicines in recent years has generated considerable attention from media and concerned parties. High-cost therapies that charge a full upfront payment can have a substantial impact on healthcare budgets, even if these costs are justified in the long run. This is particularly relevant for ATMPs, which need to secure a high reimbursed price in order to be commercially viable, due to their high costs of R&D, manufacturing and delivery. This challenge is reflected in the commercial outcomes with the ATMPs licensed in the EU so far; all eight ATMPs that have gained marketing authorisation through the European Medicines Agency (EMA) have had considerable difficulty in obtaining reimbursement in Europe [5]. No ATMP has so far achieved widespread reimbursement and access across the five biggest European countries (France, Germany, Italy, Spain and the UK), and four products (ChondroCelect®, MACI®, Provenge® and Glybera®) have been withdrawn from the market, largely due to challenges with securing reimbursement [5,6]. Furthermore, among the products still on the market, reimbursed use has been limited and patchy: Imlygic® is so far only reimbursed in Germany and the UK [7,8], and reimbursement is restricted beyond its regulatory label in the UK; Holoclar® is only reimbursed in France and Italy [5,9,10]; Strimvelis® is only reimbursed in Italy, and its use is limited in one centre in all of Europe^1^ ^1^Non-Italian patients will be required to travel to Italy for treatment (pending successfully completed P&R negotiations in their respective countries). [8]; and Zalmoxis® (which has a conditional marketing authorisation from the EMA) aimed to initiate pricing and reimbursement (P&R) negotiations in the first half of 2017 [11].

While the commercial circumstances surrounding these ATMPs differ, all of them face two common challenges: firstly, to convince payers of their value for money, and secondly to ensure that healthcare budget holders can afford to facilitate their adoption [5,8]. These challenges are exacerbated by the different perspectives and value drivers considered by various decision-makers at national versus regional and/or local levels.

In England, P&R decisions for pharmaceuticals are made by the Department of Health at the national level, based on the health technology assessment (HTA) and recommendations of the National Institute for Health and Care Excellence (NICE). NICE’s technology appraisal (TA) methodology is based on the cost-utility analysis (CUA) framework, which rewards improvements in patient survival and quality of life (QoL). The CUA considers the lifetime costs and benefits (expressed as quality-adjusted life years [QALYs]) of a new therapy and compares them to the existing standard of care (SOC). NICE deems a new therapy to be cost-effective, and recommends it for reimbursement in the NHS, if the incremental (additional) cost per additional QALY generated by the new therapy is between £20,000 and £30,000 (depending on the degree of certainty in the results, how adequately QoL has been captured and how innovative the therapy is), or below [12]. Put differently, the CUA methodology rewards innovation through a stated willingness to pay of £20,000–30,000 per additional QALY generated by a new therapy, thus incentivising manufacturers to develop therapies that improve patients’ mortality and/or QoL. For therapies that target patients at the end of their lives, this willingness to pay per QALY is increased to £50,000; it increases further and up to a maximum of £300,000 per additional QALY for treatments that provide large QALY improvements in very rare diseases (assessed under the Highly Specialised Technologies programme) [13,14].

The CUA framework assesses value for money on a per-patient basis, and allows decision-makers to prioritise the use of taxpayer money among different therapy areas to maximise patient benefit and ensure equitable access to care [15]. However, it does not consider the aggregate impact on the healthcare budget of treating the total eligible patient population with a new therapy. Budget impact analysis (BIA) is used to estimate the likely change in expenditure associated with reimbursing a new healthcare intervention at the population level. The BIA is usually calculated over a period of one to five years – either at a national level, or at a regional or local level. In contrast to the CUA, which estimates value for money on a per-patient basis, the BIA assesses affordability on an aggregate level by taking into account the population size, patient eligibility, speed of uptake and market share of the intervention [16].

Table 1 summarises some of the key differences between BIA and CUA [17].

**Table 1.** Key methodological differences between cost-utility and budget impact analyses.

	Cost-utility	Budget impact
Scope of analysis	Patient level	Population level
Main value drivers	Change in costs*	Change in costs*
	Change in patient benefits (QALYs)*	
Time horizon	Lifetime	Budget cycle or short-to-mediumterm (1–5 years)

*Compared with the SOC.

These differences in scope and value drivers create a challenge for NHS commissioners (payers) who need to ensure affordability at the population level. On the one hand, commissioners have a statutory responsibility to make funding available for a drug or treatment recommended by NICE, and that patient access is ‘not […] impeded by national or local funding or formulary restrictions, or other health system or process barrier’ [18]. On the other hand, commissioners’ budgets are not automatically increased to pay for the additional QALYs generated, which means that funds may have to be reprioritised and reallocated to allow adoption of new therapies [19].

### Addressing the affordability challenge

Several measures exist that aim to address the affordability challenge created by high-cost therapies, and to reduce payer uncertainty in reimbursing them. Similarly, there are various classifications to define and categorise these measures; however, some of them overlap, and not all are mutually exclusive. NICE’s Decision Support Unit (DSU) published a comprehensive taxonomy of different approaches in 2016, under the umbrella term ‘managed entry agreements’ (MEAs) [20], which are defined as:

‘an arrangement between a manufacturer and payer/provider that enables access to (coverage or reimbursement of) a health technology subject to specific conditions. These arrangements can use a variety of mechanisms to address uncertainty about the performance of technologies or to manage the adoption of technologies in order to maximise their effective use, or limit their budget impact’ [21].

In the following, we build on the classifications described by NICE’s DSU, as well as additional measures detailed by Marsden et al. (2017) [22].

### Cost-based MEAs (discounts and expenditure caps)

Cost-based MEAs are used to reduce the financial uncertainty surrounding the introduction of a new therapy, and typically reduce the price (through simple discounts, or by providing a certain number of treatment cycles for free) or set a total budget restriction (at the patient level, at the product level or at the therapy class level) [22]. In England, Patient Access Schemes are commonly used to improve the cost-effectiveness and reduce the budget impact of new treatments by applying confidential discounts to their list price [23]. In April 2017, a net budget impact limit (or test) was introduced [18], as described in more detail below.

While these measures improve payers’ ability to forecast and curb future expenditure, they have a negative effect on the revenue potential of innovative, highly effective therapies, such as in the case of curative ATMPs. This creates a disincentive for manufacturers to research and develop breakthrough therapies rather than therapies that deliver incremental improvements, as the reward for the longer-term benefits is diminished.

### Volume-based MEAs (restriction to the highest-value patient groups)

Limiting the number of patients eligible for treatment is another frequently applied mechanism to improve affordability. In England, NICE can restrict high-value therapies to easily identifiable patient subgroups for which the therapy meets the cost-effectiveness threshold, based on subpopulation analysis and clinical considerations. Use in a broader population can be considered subsequently when the cost-effectiveness argument can be substantiated, such as when more safety and efficacy data become available, when follow-on therapy class competition reduces prices or when products go off patent [22].

This creates an incentive for manufacturers to invest R&D in areas where the therapeutic benefit is maximised; however, this can also reduce the return on investment if patient volumes are diminished, as well as make it increasingly difficult to recruit patients for pivotal trials [22].

### Outcomes-based MEAs

Outcomes-based MEAs, commonly also referred to as ‘risk-sharing agreements’, is a group term that applies to a range of reimbursement approaches that aim to ensure rapid access to new therapies, obtain best value for money and ensure affordability [21]. These MEAs tie manufacturers’ compensation to defined clinical outcomes (milestones), and come in different forms, including money-back guarantees (e.g., rebates in the case of treatment failure, relapse, etc.), conditional treatment continuation (e.g., continued treatment of responding patients only) or a price that is linked to outcomes [20] (e.g., proportion of patients responding).

Outcomes-based MEAs reduce payers’ uncertainty around the clinical outcomes of the therapies, while allowing manufacturers to be remunerated for the value that their products actually deliver. However, the application of outcomes-based MEAs has been limited due to the administrative burden of executing them, both in terms of defining the outcome milestones and in terms of collecting the data [22]. This is especially true for conventional pharmaceuticals (e.g., orals), but this may not constitute too much of an additional hurdle for ATMPs, as many of them are already required by regulators to track patient outcomes in registries [24].

Another type of outcomes-based MEA is for a therapy to be reimbursed at the time when the health outcome occurs rather than at the time of treatment (e.g., per year that a patient remains free of disease). This option is highlighted by both the NICE DSU and in NICE’s regenerative medicine study (a TA exercise for a hypothetical chimeric antigen receptor T-cell therapy in relapsed or refractory B-cell acute lymphoblastic leukaemia) as a potential way to address the affordability issues related to therapies with long-term benefits and a short duration of treatment administration, such as ATMPs [20,25]. Annuity payments is one such mechanism, whereby a constant amount of money is paid to manufacturers per year for a specified period of time (or in perpetuity) [26]. Under this arrangement, high-value, one-off therapies are paid for as if they were ongoing treatments, rather than charging the full amount at the time of administration [25]. This reduces the annual budget impact for payers, as well as the uncertainty around long-term performance and value, as payments can be discontinued if the patient does not sustain the desired response. This means that the manufacturer assumes the risk associated with the uncertainty around longer-term claims, and that the potential long-term value of a one-off ATMP would not need to be compromised due to short-term budgetary concerns.

### Other financial instruments (reinsurance and amortisation)

Reinsurance and amortisation are measures that apply financial instruments to help payers manage their short-term financial exposure, which have been suggested as a potential solution to the affordability challenge [22].

Reinsurance is a measure applied in the United States today whereby payers insure themselves against payouts that could not have been predicted and which would threaten their financial stability. One potential case is where an insurer experiences a high number of patients requiring very costly treatments in a very rare disease area. However, recent experience suggests that some reinsurers are considering excluding gene therapies from reinsurance policies, which would limit their potential application [22].

Amortisation is another measure whereby payers can take up a loan to cover the high upfront costs of a new therapy, and subsequently repay the amount over a longer period of time (e.g., over the period of the expected benefits), which spreads the financial impact of the upfront cost over several years. An example of this is seen in Spain, where the national government provided low-interest loans to regional health authorities to pay for high-cost hepatitis C treatments [22].

While such financial instruments make payers’ annual budget impact more manageable, they also mean that the manufacturers do not assume the risk stemming from the uncertainty around long-term benefits and value of the product, as these arrangements are between the payers and third parties, rather than between payers and manufacturers. This means that such arrangements would be more acceptable for payers when therapies with more certain outcomes are concerned.

### England’s net budget impact test

In an effort to address the discrepancies between the prices deemed cost-effective on a per-patient basis, and the aggregate impact high-cost therapies have on the NHS budget, NICE published a joint consultation with NHS England in October 2016, proposing to introduce a net budget impact threshold to the NICE TA and Highly Specialised Technologies programmes [27]. More specifically, the consultation reads:

● Having considered the frequency and magnitude of high budget impact NICE-recommended technologies, NHS England proposes to set the threshold at £20 million per annum.● The threshold would be regarded as having been triggered if it is projected to be reached or exceeded in any of the first 3 financial years of its use in the NHS.

Following public consultation, this proposal was approved by NICE’s board in March 2017, and a net budget impact test now applies to evidence submissions made to NICE after 1 April 2017, using the proposed £20 million threshold as the trigger value [13]. Therapies that are anticipated to exceed this threshold, and for which an agreement is not reached in order to bring the annual net budget impact below it, are subject to a ‘phased’ introduction, typically over three years, to help manage the NHS budget impact [13]. A procedure is outlined for varying the funding requirements under the phased introduction [4], but no information is available yet to indicate what form these funding variations might take.

Although NICE has stated that the £20 million test is ‘not necessarily the maximum amount that the NHS would commit to funding a new technology in any one financial year’ [27,28], it establishes an expectation and requirement for manufacturers to comply with when launching a new therapy in England, and is therefore a mechanism that deserves to be considered in more detail.

## Methods

Net budget impact is defined as an estimation of the change in the use of a healthcare technology, or the introduction of a new one, and is a forecast of rates of use (or changes in rates of use) with their consequent short- and medium-term effects on budgets [26]. In accordance with the budget impact test definition, we apply annual net budget impact calculations in our example, as defined in Equations (1 and 2).

Annual net budget impact per patient

(1)
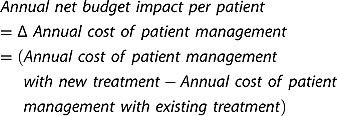

Annual net budget impact (total)

(2)
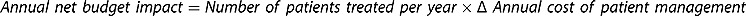

Because our example focuses on maximising patient access (i.e., the maximum number of patients that can be treated per year without exceeding the £20 million budget impact threshold), we solve the annual net budget impact equation for the number of patients treated, as shown in Equation (3). By dividing the £20 million threshold value by the annual net budget impact per patient, we elicit the number of patients that can be treated before the budget impact test is exceeded.

Maximum number of patients eligible for treatment without exceeding the net budget impact test

(3)
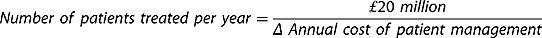

Two levers can be applied to avoid triggering the budget impact threshold: a reduction in price and/or a reduction in patient volumes. Our example uses the introduction of a hypothetical ATMP to explore the annual net budget impact per patient and the resulting constraints on patient volumes, compared with the SOC (as detailed in Table 2).

**Table 2.** Key features of (the hypothetical) Product X and standard of care.

	Product X (ATMP)	Standard of care
Treatment regimen	One-off treatment	Chronic treatment
Duration of effect	3 years*	Short-term (requires ongoingreadministration to maintain effect)
Cost of therapy	£20,000**	£1700 per year

*Subject to uncertainty.

**Includes the cost of administration and any other healthcare services needed.

We explore how patient access differs under the budget impact test depending on the type of payment scheme applied. We detail results for (1) where a full upfront payment is used, and (2) where an annuity-based payment scheme is used, under which the product value is spread over the assumed duration of the effect – i.e., £20,000 over three years. The annuity-based payment scheme was chosen because it is an increasingly relevant method to examine given the interest shown in it by both the NICE DSU report and NICE’s regenerative medicine study as a potential payment method for sharing and managing financial risk in the case of high-cost ATMPs [20,25].

Our findings detail firstly how the annual net budget impact per patient differs over the first three years (i.e., the scope of the budget impact test), according to whether a full upfront or annuity-based payment scheme is applied. We display the results for each year individually, as well as the total, three-year budget impact (it is worth noting that the costs in Years 2 and 3 are not adjusted for inflation, as there is no information or precedent in the public domain that guides on how such adjustments should be made under the budget impact test).

Subsequently, we explore two additional scenarios where the duration of effect and product value of Product X is increased, as well as the price that is (hypothetically) found cost-effective by NICE, to see how annuity-based payments could impact patient access as Product X’s benefits and value increase. The additional scenarios tested are: five and 10 years’ duration of effect at a cost-effective price of £30,000 and £50,000, respectively (see Table 3).

**Table 3.** Scenarios of duration of effect and cost of patient management (over the duration of effect) for (the hypothetical) Product X.

	Duration of effect	Cost of therapy
Scenario 1	3 years*	£20,000
Scenario 2	5 years*	£30,000
Scenario 3	10 years*	£50,000

*Subject to uncertainty.

## Findings

**Figure 1.**
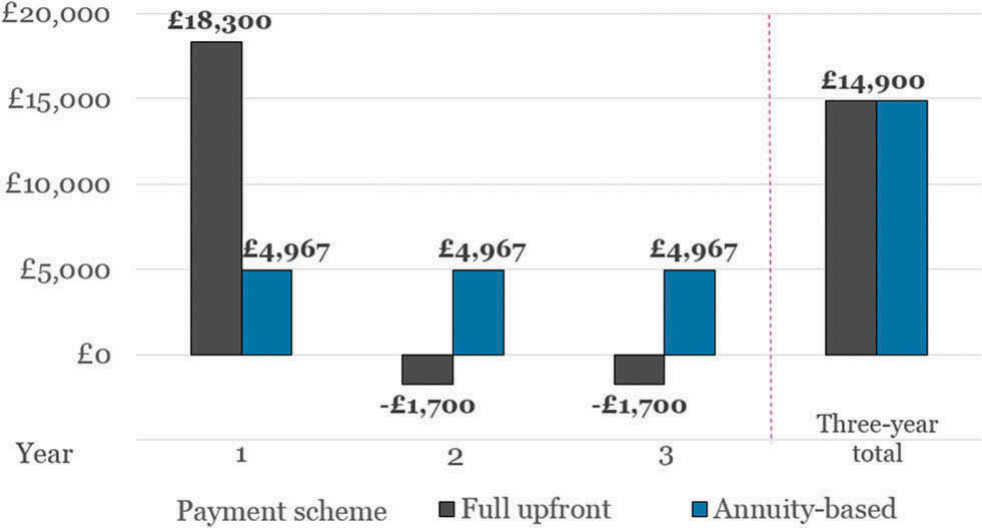
Annual and total net budget impact per patient over the first three years, according to payment scheme (i.e., full upfront and annuity-based payments).

Based on the assumptions and scenarios outlined above, Figure 1 illustrates how a full upfront payment for Product X results in a net budget impact per patient of £18,300 in Year 1, while annuity-based payments result in a uniform budget impact per patient of £4967 in each of the three years.

When comparing the two payment schemes, it is apparent that the total net budget impact over the course of the three years is the same for both (£14,900). However, since the number of patients eligible for treatment under the budget impact test is determined by the highest net budget impact in any one year *individually*, the maximum number of new patients that can be treated using a full upfront or annuity-based payment is based on the £18,300 and £4967 figures, respectively (in accordance with Equation 3).

**Figure 2.**
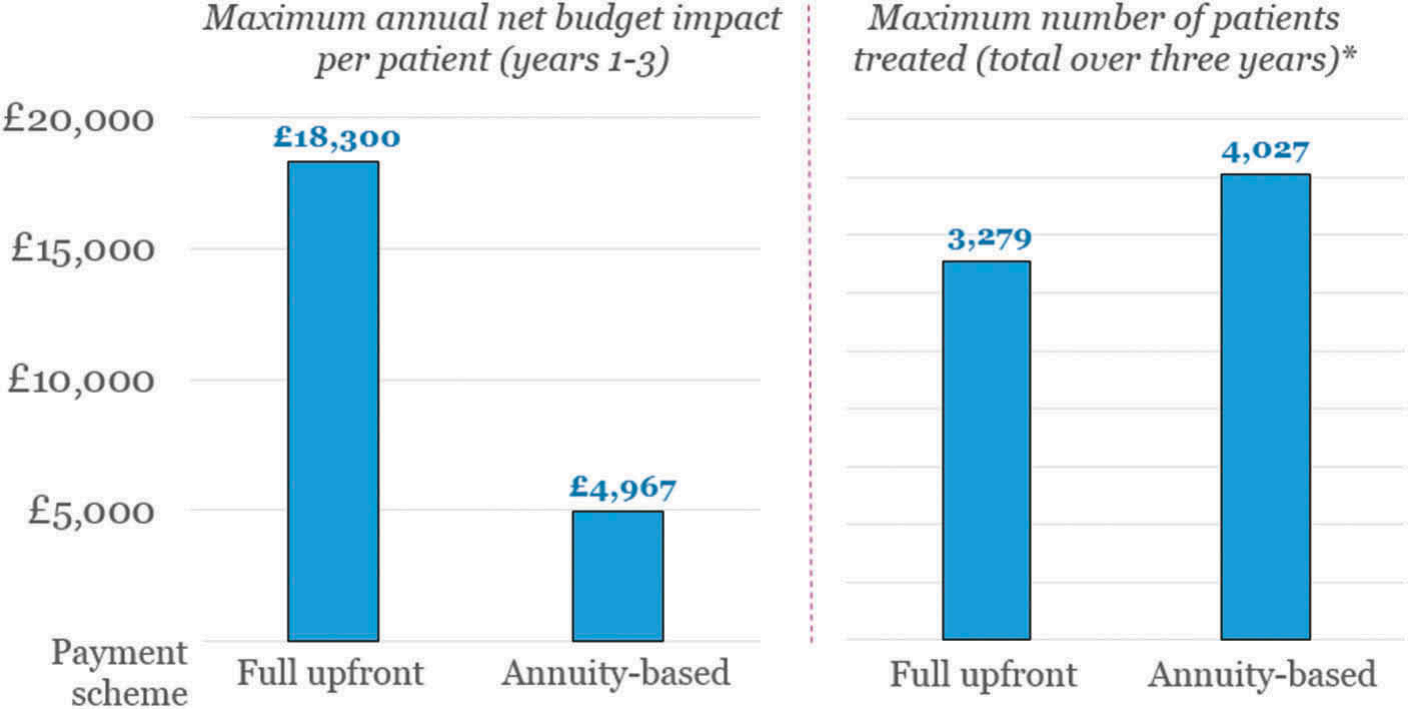
Annual net budget impact per patient in Years 1–3, and corresponding numbers of patients eligible for treatment,* according to payment scheme (i.e., full upfront or annuity-based payments). *While meeting the requirements of the £20 million budget impact test.

In the left-hand side of Figure 2, we illustrate the maximum annual net budget impact per patient as incurred by the NHS in any of the first three years after launch, and in the right-hand side, the corresponding patient numbers eligible for treatment without exceeding the £20 million threshold, over three years (i.e., the claimed duration of the treatment’s effect). We display results separately depending on the payment scheme applied – that is, full upfront or annuity-based payments.

A maximum net budget impact per patient of £18,300 means that the budget impact test limits the eligible patient population to a maximum of 1093 patients treated per year, or a maximum of 3279 over a three-year period. If an annuity-based payment scheme were adopted instead, the maximum number of patients eligible for treatment over these three years would be increased by 23%, to 4027.

The annuity-based payment mechanism increases the number of patients eligible for treatment because it spreads the cost of the therapy over several years (in this example, over the duration of effect; i.e., three years), hence reducing the cost of treatment in any one year. Also, the lower annual cost of Product X means that the cost of the standard of care (i.e., £1700 per year) has a proportionally greater offsetting effect on the annual net budget impact per patient (as compared with a full upfront payment), which enables more patients to be treated without exceeding the £20 million threshold.

In Table 4, we detail the maximum number of patients that can be treated with Product X in each of the three years after launch (without exceeding the budget impact test), based on the net budget impact results shown in Figure 1.

**Table 4.** Maximum number of patients eligible for treatment* according to the annual budget impact per patient.

Year	Full upfront payment	Annuity-based payments
1	1093	1342**
2	1093	1342**
3	1093	1342**
Total over three years	3279	4027

*Eligible for treatment with Product X, in order to meet the requirements of the £20 million budget impact test. **Assuming that a similar number of patients are initiated in each year (i.e., 4027 divided by three).

The annuity-based payment scheme not only considerably increases the total number of patients eligible for treatment over the three years, but also allows decision-makers greater flexibility in how to manage the number of patients treated per year. In fact, it would be possible to treat far more than 1342 – e.g., in Year 1 – and still meet the requirements of the net budget impact test, as long as the total number of patients treated in the first three years does not exceed 4027^2^ ^2^Theoretically, all 4027 patients could be treated in Year 1; however, this would mean that no additional patients could initiate treatment in Years 2 and 3 (without discontinuing payments for patients who have already been treated with Product X), which is hardly realistic or desirable.; this would be particularly useful in indications where there is a large proportion of prevalent patients, and where the incidence is low, such as patients with a rare disease where the life expectancy is long. In such cases, annuity-based payments would allow the NHS to treat more of the prevalent population at launch, and then focus on the incident population in subsequent years. Alternatively, Product X could be used to treat 1342 patients in each of the three years (as shown in Table 4), and still be within the bounds of the £20 million threshold. Either way, annuity-based payments increase the number of patients that can be treated without exceeding the budget impact test threshold, as compared to using a full upfront payment.

These trends are even more pronounced if we consider scenarios where the performance of Product X is enhanced in terms of sustainability of effect, as shown in Table 3 (all other assumptions detailed in Table 2 remain the same).

**Figure 3.**
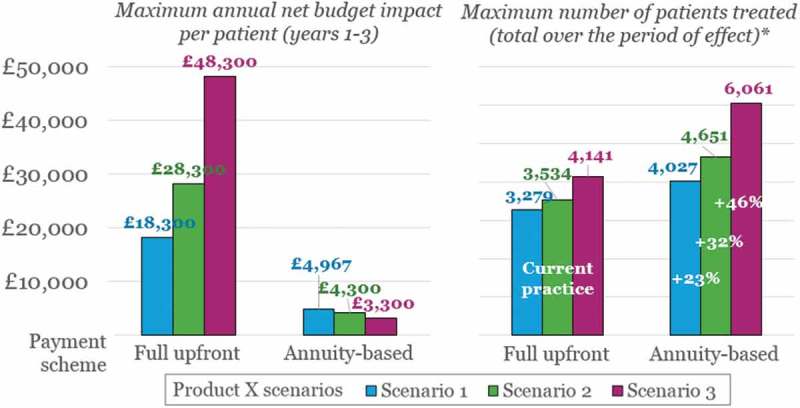
Annual net budget impact per patient in Years 1–3, and corresponding numbers of patients eligible for treatment,* according to payment scheme (i.e., full upfront or annuity-based payments). *While meeting the requirements of the £20 million budget impact test; the period of effect is three, five and 10 years for Scenarios 1, 2, and 3, respectively.

In Figure 3, we compare the results shown previously in Figure 2 (Scenario 1) to those of Scenarios 2 and 3, in terms of (1) the maximum annual net budget impact per patient in Years 1–3; and (2) the corresponding patient numbers eligible for treatment without exceeding the £20 million threshold, over the claimed duration of the effect (i.e., five and 10 years). As above, we display results separately depending on the payment scheme applied – that is, full upfront or annuity-based payments. In the case of annuity-based payments, the product cost is spread over the duration of the effect – i.e., £20,000, £30,000 or £50,000 spread over three, five or 10 years, respectively.

Figure 3 shows that applying an annuity-based payment scheme rather than full upfront payments (labelled as ‘Current practice’ in Figure 3) provides an even greater proportional increase in patient numbers as Product X’s duration of effect increases: from 23% in Scenario 1, to 32% and 46% for Scenarios 2 and 3, respectively. Again, this is due to the reduction in annual net budget impact per patient, which is driven by the extended time period over which the total therapy cost is spread.

## Discussion

The discrepancy between the cost-utility methodology (i.e., value for money on a per-patient basis) and affordability for payers is a key reason for the slow adoption of therapies with favourable NICE recommendations in England. This issue is particularly relevant for high-cost, one-off therapies (e.g., curative treatments with long-term benefits) charging a full upfront payment, because the long-term product value is frontloaded in one large payment, which causes considerable payer concerns.

Budget impact considerations are nothing new in P&R negotiations; however, what *is* new with net budget impact test is an explicitly defined threshold level for budget impact in England. This increases the transparency of the P&R process, and makes access negotiations more predictable, which in turn reduces uncertainty in manufacturers’ strategic planning and increases the confidence in commercial forecasts, both of which are valuable features from the industry point of view.

However, while the budget impact test is a more transparent way to address the affordability issue, our example shows that it also means that conventional payment arrangements (i.e., a full upfront payment at the time of treatment) can lead to suboptimal patient access. This issue is particularly relevant for new, high-value, one-off treatments, such as curative ATMPs, where the cost-effective price is driven to a greater extent by future benefits and savings accruing, rather than simply by impact on costs and benefits generated in the year of treatment.

Furthermore, the net budget impact analysis disadvantages therapies with a high impact on QoL and mortality, as these are the factors that drive the improvement in QALYs. As stated previously, NICE most commonly applies a willingness to pay of £20,000–30,000 per additional QALY generated over a lifetime; however, since this value simply represents an additional cost in the budget impact calculation, therapies that offer larger QALY gains are disproportionately disadvantaged as compared to those with lower QALY gains. This is particularly relevant for ATMPs that have the potential to greatly improve the number of QALYs enjoyed by patients over a lifetime. This challenge could be even more profound for therapies that target end-of-life or very rare diseases, where NICE currently accepts a far higher cost per additional QALY – up to £50,000 and potentially up to £300,000 respectively.

It should be noted that whereas the budget impact test in its strictest interpretation is poised to limit patient access based on cost alone (without consideration for QoL and survival), this will not be the case in the context of access negotiations in England, as it is used in conjunction with the cost-utility framework, which rewards QALY gains. Thus, if access restrictions are imposed, these will likely be to subpopulations with the greatest expected clinical and economic benefit. This creates an incentive for manufacturers to focus R&D efforts on indications and therapeutic positions where the disease burden and patient management costs are high, as the net budget impact per patient, and therefore the constraint on patient numbers would be lower; this can be favourable from a ‘room for innovation’ perspective, as these patients tend to have high unmet need, and therefore the willingness of key market access stakeholders to adopt innovative therapies for such populations is greater. It also favours targeting smaller populations like end-of-life, orphan and ultra-orphan indications, which are often underserved by therapeutic innovation. On the other hand, the net budget impact test can potentially disadvantage patients with diseases that are associated with low management costs (low economic burden), which is not necessarily synonymous with a low clinical burden or unmet need.

Our example shows that annuity-based payments provide a solution that can improve patient access under the net budget impact test, and also that this payment scheme is well poised to tackle the challenge of treating a greater number of patients in the year of launch (which is particularly relevant in indications where the prevalent population is much larger than the incident population). Additionally, if future payments are made conditional on maintaining (or achieving) a defined clinical outcome, they also help distribute financial risk from NHS payers to manufacturers, and reduce the uncertainty around real-life product performance and long-term effectiveness, which is a common challenge in their P&R negotiations. This is echoed in NICE’s regenerative medicine study (the hypothetical TA exercise), which explores how annuity-based payments (‘leasing’) and outcomes-based MEAs can reduce payer uncertainty and thereby increase the likelihood of a product being recommended for reimbursement.

An outcomes-based MEA using annuity payments provides a means to address both the consequences of decision uncertainty, as described in the NICE regenerative medicines study [25], and the budget impact test, as described by NICE and NHS England [4]. This would reward manufacturers for developing therapies that truly provide long-term benefits, while mitigating payer risk and increasing patient access to therapeutic innovation at launch, without exceeding the net budget impact test. This is an opportunity that should be seized by manufacturers and the NHS alike when considering options for the phased introduction of high-value therapies.
